# Study on Fatigue Behavior and Fracture Mechanism of LMD Ti-6.5Al-3.5Mo-1.5Zr-0.3Si Alloy Based on Microstructure

**DOI:** 10.3390/ma17246112

**Published:** 2024-12-13

**Authors:** Yuxue Wu, Yongxin Wang, Yunmei Lu, Chenxi Zhao

**Affiliations:** State Key Laboratory of Solidification Processing, Northwestern Polytechnical University, No. 127, Youyi Road (West), Xi’an 710072, China; wyx0214@mail.nwpu.edu.cn (Y.W.); luyunmei@mail.nwpu.edu.cn (Y.L.); zhaochenxi@mail.nwpu.edu.cn (C.Z.)

**Keywords:** additive manufacturing, crack propagation, fatigue, properties microstructure

## Abstract

This study explores the fatigue behavior and fracture mechanisms of TC11 titanium alloy formed by laser metal deposition (LMD) and subjected to double annealing. The research focuses on how the alloy’s unique microstructure, consisting of alternating equiaxed and columnar crystals, influences its fatigue performance. The microstructure’s basket-like α’ phase, made up of both plate-shaped and needle-like structures, leads to variations in crack growth behavior, as shown in the relationship between the crack growth rate and the stress intensity. An analysis of slip patterns reveals that equiaxed crystals undergo more frequent deformation, accelerating crack propagation compared to the more evenly distributed deformation in columnar crystals. These findings suggest a new approach for improving the fatigue resistance of 3D-printed titanium alloys by optimizing their microstructure. This study provides valuable insights for enhancing material toughness and extending the lifespan of titanium alloys in applications such as aerospace and biomedical engineering.

## 1. Introduction

Ti-6.5Al-3.5Mo-l.5Zr-0.3Si Chinese grade TC11 is one of the typical dual-phase titanium alloys, which has high room temperature strength, excellent corrosion resistance, and hot workability [1,2,3]. However, the characteristics of titanium alloy determine that the alloy components have many production processes, poor processability, and high processing costs. The material utilization rate of forged parts is often less than 10% [4,5]. In addition, titanium alloy has a large flow stress and needs to be formed under high-temperature conditions, and which aggravates the cost. Various factors restrict the development of TC11 in the aviation field. Additively manufactured (AM) titanium alloys, which can meet the efficient production in small batches or components with complex geometries, thus have potential uses in aerospace, biomedical, and nuclear industries [6,7]. Some components are subject to fatigue loading in use, and the fatigue behavior study of high-cycle loading cycles has become a new field of fatigue research to meet the demand for the long fatigue life of service components. Similarly to conventionally manufactured metal materials for forgings, AM titanium alloys may still fail at fatigue cycles greater than the conventional fatigue limit 10^7^ [8]. Laser melting deposition (LMD) technology is a forming method to build complex metal parts by adding metal powder or wire layer by layer [9]. Compared with forgings, LMD technology has the advantages of high design freedom, short production cycle, and high material utilization rate, and has attracted the attention of many scholars in the field of mechanical properties research [10,11,12]. Therefore, it is necessary to study the fatigue properties of LMD TC11, including fatigue life and fracture mechanism.

Fatigue failure is a major concern in the aviation industry, affecting critical components such as aircraft fuselages, turbine blades, and gas turbines, all of which are exposed to cyclic stress. The fatigue performance of titanium alloys is strongly influenced by their microstructure [13,14,15]. Previous research has shown that the mechanical properties of the titanium alloys produced by laser metal deposition (LMD), such as Ti-6Al-4V, are significantly affected by the microstructure formed during the manufacturing process [16,17]. Additionally, studies have identified that variations in the orientation and size of α-phase structures near crack-initiation defects play a key role in the fatigue life of additively manufactured titanium alloys [18].

While much has been studied on the fatigue behavior of laser-formed titanium alloys, there are still gaps in understanding how specific microstructural features influence fatigue mechanisms. One such feature is the unique bimodal microstructure of LMD TC11, which includes alternating regions of equiaxed and columnar grains [19], along with the basketweave pattern of the martensitic α’ and primary α phases [20]. Although the formation of this microstructure has been explored [21], the role of columnar grain boundaries as sites for crack propagation and the mechanisms behind crack initiation and growth are not fully understood.

In this study, we investigated the fatigue fracture behavior of double-annealed LMD TC11 titanium alloy, focusing on how its unique bimodal microstructure influenced fatigue performance. Similarly to Azarniya et al.’s work, we found that the microstructure formed during additive manufacturing significantly affected the mechanical properties [22], but our study further revealed that columnar grain boundaries act as preferential sites for crack propagation, a factor not fully explored in previous research. Compared to Shamir et al.’s findings, which highlighted the role of α-phase orientation in fatigue life dispersion [23], our research emphasizes how the interaction between equiaxed grains and columnar grain boundaries accelerates crack propagation, providing a more complex understanding of fatigue behavior. Unlike other studies that focus on heat treatment’s overall impact on mechanical properties, our work specifically links microstructural features (such as the α’ phase and grain boundary structure) to crack growth mechanisms. Overall, this study fills a gap in the literature by connecting microstructural characteristics with fatigue behavior, offering new insights for improving the fatigue performance of additively manufactured titanium alloys, particularly in high-performance applications like aerospace.

## 2. Experimental Procedure

### 2.1. Laser Melting Deposition

TC11 titanium alloy spherical powders with an average size of 60–200 mesh, prepared by plasma rotating electrode atomization and supplied by Jiangsu Metalink Special Alloys Corporation (Nanjing, China), were used as feedstock materials. The chemical composition of the alloy powders, analyzed using inductively coupled plasma mass spectrometry (ICP-MS), is presented in Table 1. The alloy powders were subsequently loaded into a commercial PBF-LB machine (iDEN160) equipped with a Yb-fiber laser source. The spherical powder of the TC11 titanium alloy conveyed coaxially was melted and subsequently solidified in a molten pool formed by a moving high-energy laser beam spot in a forming chamber filled with argon gas. The scanning thickness of a single layer was 2 mm to 3.5 mm. The single layer was scanned back and forth, and a thick plate with dimensions of 34 mm × 300 mm × 230 mm was finally formed by repeatedly lap depositing a new layer on the deposited layer. The process parameters of the laser melting deposition were as follows: the laser power is 4–6 kW, the spot diameter is 5mm, the scanning speed is 800–1000 mm/min, the lap rate between tracks is 45%, the powder feeding speed is about 500 g/h, and the oxygen content is less than 5 × 10^−5^ (volume fraction).

**Figure 1:**
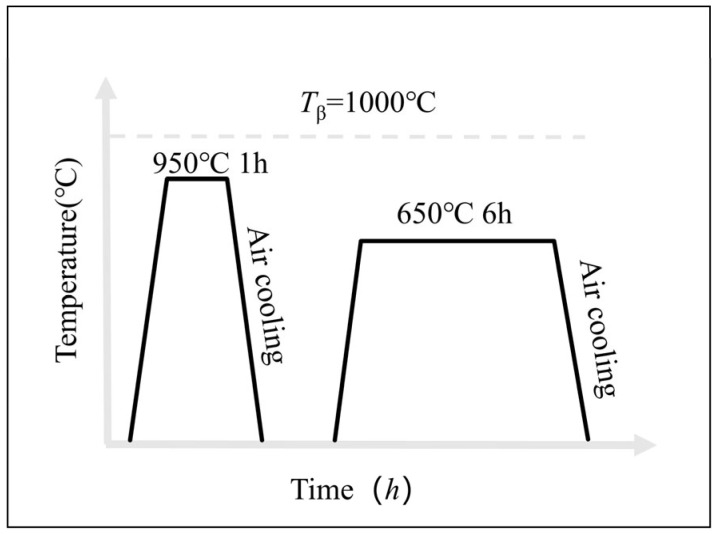
Heat treatment process of deposited specimen.

Studies have shown that heat treatment is often used to change the microstructure to improve fatigue properties and plasticity [9,24]. Referring to the heat treatment process of TC11 forgings, some deposited plates were subjected to subsequent double-annealing heat treatment. After molding, the LMD TC11 samples were double annealed by a heat treatment system. The first annealing was held at 950 °C for one h, then air-cooled, and the second annealing was reheated to 650 °C for six h so as to relieve stress and stabilize the microstructure. The selection of these specific annealing parameters was based on findings from previous studies, which highlight the importance of temperature and duration in optimizing the microstructure and mechanical properties of TC11 titanium alloys. In particular, research has shown that the deposited structure of TC11 titanium alloy exhibits a columnar/equiaxed alternate growth pattern along the deposition direction, primarily consisting of the Widmanstätten structure and mesh basketweave structure, with α phase clusters along the grain boundaries and intra-granular acicular α phase. The annealing temperature significantly affects the refinement of the intragranular α phase and the formation of continuous α_GB_ phases, as well as the transition to a refined mesh basketweave structure. A first annealing temperature of 950 °C and a duration of one hour were chosen to allow sufficient dissolution of the intragranular α phase and to promote the formation of a more uniform microstructure without excessive grain coarsening. This finding is consistent with those of previous studies in this field [12] which indicate that as the annealing temperature increases in the dual-phase zone, the intragranular α phase becomes finer, and the grain boundary α phase spheroidizes and becomes discontinuous at higher temperatures. The second annealing at 650 °C for six hours aims to relieve residual stresses from the LMD process and stabilize the microstructure, improving both fatigue strength and plasticity. The extended annealing time at 650 °C ensures a balance between strength and ductility, which is crucial for achieving the desired mechanical properties. The heat treatment process is depicted in Figure 1.

### 2.2. Fatigue Performance and Crack Growth Test

**Figure 2:**
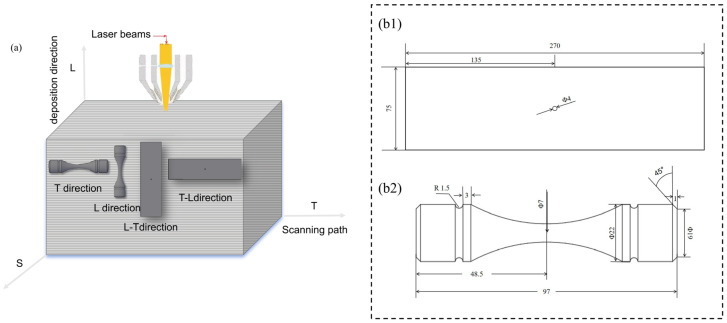
(**a**) Schematic illustration of the deposition direction. Schematic representation of specimens: (**b1**) Fatigue crack extension specimen, (**b2**) Fatigue tensile specimen.

In order to test the crack growth rate and fatigue properties, the obtained plates were made into corresponding samples. In order to distinguish different additive directions in order to study the difference in their properties, three forming directions were defined: the deposition direction was the L direction, the laser scanning direction was the T direction, and the vertical laser scanning direction was the S direction. The orientation of each sample is defined as shown in Figure 2a.

In the crack growth rate test, the da/dN~ΔK relationship curve of the two crack growth surfaces of the materials L-T and T-L was measured. The test environment was 3.5% NaCl solution at room temperature, the stress ratio of R-1 was selected, and the test sample was an M (T) sample with b = 4mm, W = 75mm, and L = 270mm. The sample size is shown in Figure 2(b1).

The material orientation of the axial loading fatigue test was in the L and T directions. The test specimen, Kt = 1, is shown in Figure 2(b2), and the stress ratio was R = −1. The group method was used to measure the S~N curve data. A group of samples were made at each stress level. The number of samples in each group depended on the dispersion of the experimental data and the requirement of 95% confidence. For the determination of the fatigue limit, the lifting method was adopted, and the stress level of lifting was generally level 4. The stress level of the first specimen was slightly higher than the predicted fatigue limit. We determined the test stress level (decrease or increase) of the next specimen according to the test result (failure or pass) of the previous specimen until all the tests were completed.

The fatigue test part used a Qianbang QBG-50 high-frequency fatigue testing machine, manufactured by Changchun Qianbang Testing Equipment Co., Ltd., Changchun, China, which adopted stress control, sine wave, banner loading, and the frequency range was 120–130 Hz. The number of breaking cycles was taken as its fatigue life. If it exceeded 10^7^ without breaking, it would not fail at this stress level.

### 2.3. Microscopic Morphology Test

The fracture morphology was observed by a TESCAN VEGA3 LMH scanning electron microscope, manufactured by TESCAN Co., Ltd., Shanghai, China and the failure analysis was made by cutting the fracture crack source diameter of the TC11 titanium alloy fatigue specimen with a thickness of 2 mm and a length of 1.8 mm. The surface was polished with 400 mesh, 800 mesh, 1200 mesh, 2000 mesh, 3000 mesh, and 5000 mesh sandpaper, mechanically polished for 30 min, and then vibration polished for 8 h to make the surface smooth. The samples were characterized using a TESCAN VEGA3 LMH scanning electron microscope equipped with an Oxford diffraction (EBSD) accessory to characterize the local crystal orientation (represented using an IPF diagram) and the local plastic deformation (illustrated using a KAM diagram). The surface was etched using Kroll’s reagent (volume ratio of HF, HNO_3_, H_2_O of 1:2:7) and subsequently observed using an LWT300LPT polarizing microscope, manufactured by Beijing Cap High Technology Co., Ltd., Beijing, China.

## 3. Results and Discussion

### 3.1. Crack Initiation

**Figure 3:**
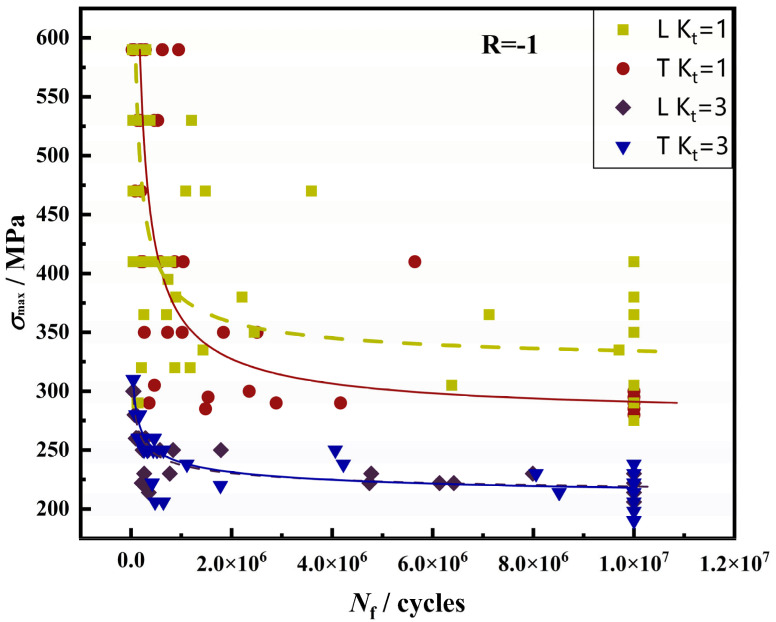
Fitting diagram of S-N curves in two different forming directions of Kt = 1 and Kt = 3.

The S-N fitting curves of two different forming directions of Kt = 1 and Kt = 3 are shown in Figure 3. Due to stress concentration and other factors, the fatigue life of the notched samples was hardly affected by the microstructure, while the microstructure and microscopic defects in the smooth samples were the main factors of fatigue performance. Moreover, the data results in the S-N curve show that the variation coefficient and standard deviation in the L direction were larger than those in the T direction, so its dispersion was larger, and the stress level was lower. It can be judged that the fatigue performance of the L direction was worse than that in the T direction. The lives of the two samples were N_f_ = 53.3 × 10^3^ (denoted as T1 sample) and N_f_ = 623.8 × 10^3^ (denoted as T2 sample), respectively.

**Figure 4:**
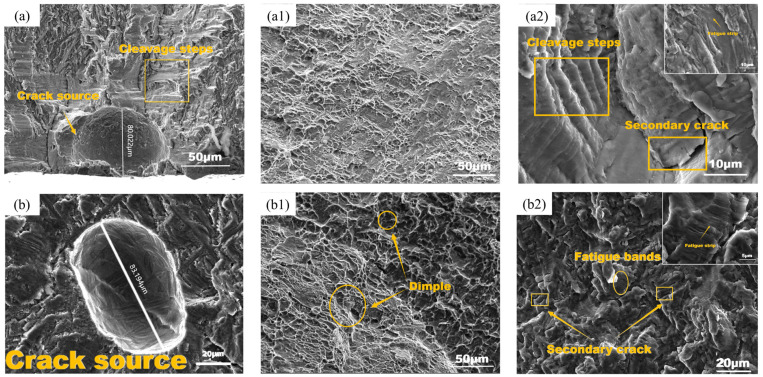
T1 sample crack source morphology (**a**); dimple morphology (**a1**); stable propagation zone morphology (**a2**); T2 sample crack source morphology (**b**); dimple morphology (**b1**); stable propagation zone morphology (**b2**).

The local SEM diagram of the fracture surface of the two samples after fatigue tension is shown in Figure 4. The macroscopic fracture surface of the fatigue sample was mainly divided into three parts: fatigue source region I, crack stable propagation region II, and instantaneous fracture region III. According to the report, the laser-formed fatigue crack of TC11 originated from the pores near the surface, and most of these pores were located at the columnar grain boundaries during solidification. The size of the pores and their distance from the surface were two important factors to control the fatigue life of the specimens. The larger the pores or the closer they were to the surface, the shorter the fatigue life [10,22,25,26]. In this experiment, there were many hole defects in the LMD TC11 after heat treatment, and there were many possibilities for their formation, such as the pores that came with the powder, some chemical reactions that may have produced gasses during laser processing, and inappropriate molding parameters causing the bubbles in the molten pool to be unable to escape before the molten material solidified. During the melt solidification process, the shielding gas entered the melt material to form pores [27]. The fracture morphology of the two samples with different lives was radioactively extended outward with hole defects at the center. It could be seen that these holes were the initial position of fatigue fracture, that is, the crack source, which may have been due to local “softening” or stress concentration caused by the uneven pores. Under the action of continuous cyclic loading stress, violent plastic flow occurred around the pores, which led to crack nucleation. The size of the crack source hole in the low-life specimen (S ≈ 11,588.94 μm^2^) was smaller than that in the high-life specimen (S ≈ 3583.37 μm^2^), but the difference was that the crack source hole in the low-life specimen was a semicircular hole on the surface, and the high-life crack source hole was a subsurface elliptical hole. It could be seen that surface hole defects were more likely to cause cracks than internal defects, regardless of the size of the hole as the source of crack formation.

There were a large number of slip steps with small spacing and basically parallel fatigue marks on the surfaces in the expansion areas of both specimens. Each fatigue grain represented the forward propagation distance of the fatigue crack under an alternating load cycle. The spacing between these striations reflected the crack growth increment per cycle, which was closely related to the stress intensity range (ΔK) and the local microstructural properties. The difference between the two was also obvious. The short-life sample had a large number of obvious cleavage steps and river patterns, which were typical cleavage fracture characteristics, while the long-life sample had obvious dimples and tear edges in the third stage, which showed that the fracture process was mainly ductile fracture with obvious plastic deformation. It was preliminarily judged that the fracture toughness of the latter was obviously higher than that of the former, which was one of the reasons for the difference in life between the two.

In addition, there were a large number of secondary cracks and tear edges in the crack propagation zone, indicating that it had the characteristics of transgranular cracking. In order to explore the influence of secondary cracks on fatigue life, the fractures of four samples with the same stress of 590 Mpa were selected for observation, and the number of secondary cracks in the area along the diameter of the crack source under a 300 times electron microscope (as shown in Table 2) was counted. It could be seen from the statistical table that the content of secondary cracks in the high-life samples also increased accordingly, so the existence of these secondary cracks was helpful to consume the energy generated by stress and improve the fatigue life of the samples.

### 3.2. Crack Propagation

**Figure 5:**
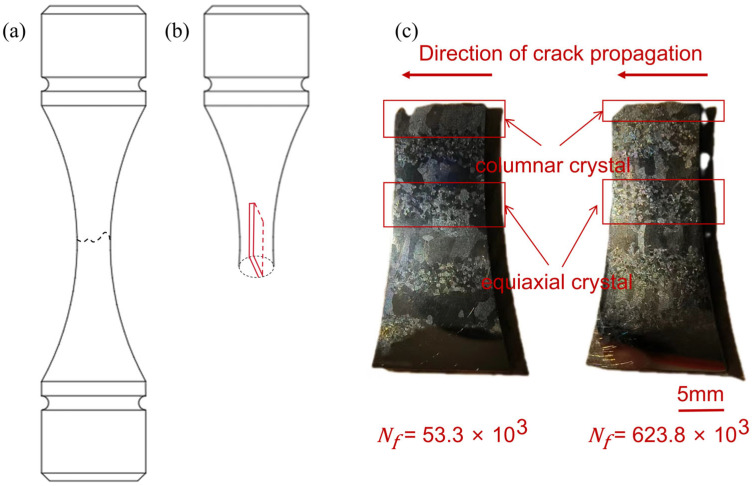
Schematic representation of the fatigue tensile fracture location (**a**); schematic representation of the wire-cut sampling (**b**); schematic representation of the macroscopic micro-morphology of the sample (**c**).

The porosity mainly affected the first stage of crack propagation, while the internal stress mainly affected the subsequent stages of crack propagation. The longitudinal cut diagram of the sample after corrosion fatigue fracture is shown in Figure 5. It can be seen that after heat treatment, LMD TC11 showed a bimodal microstructure macroscopically, and its equiaxed crystal region and columnar crystal region appeared alternately in turn. Both crystal regions showed a mesh basket microstructure with a width of 2~3 mm. Some studies say that the formation of this macroscopic morphology was related to external factors during molding. For example, the shape of the molten pool, the direction of heat dissipation, etc. [28], could be seen from the T forming direction sample, showing that the areas where both cracks were generated were columnar crystal regions, which were related to the fact that the columnar crystal regions were more likely to form pores during forming.

**Figure 6:**
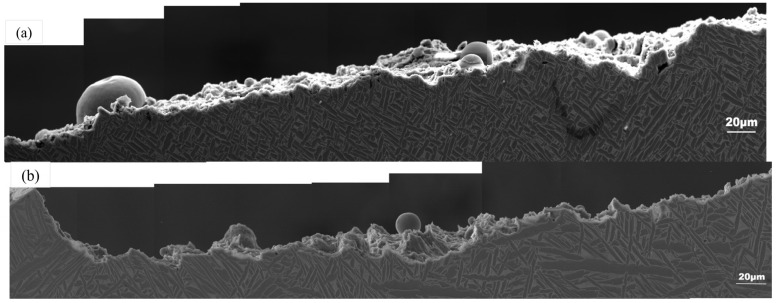
Partial SEM diagrams of T1 (**a**) and T2 (**b**) crack propagation path.

The microstructure under the SEM electron microscope is shown in Figure 6; it contained about 90% α phase and about 10% β phase, and the α phase included a lamellar αl phase and the needle-like secondary α’ transformed from β during heat treatment. The long diameter of these “root-whisker” αs was 30~50 μm, and the thickness of αl lamellae was about 15 μm. They and a very small amount of β phase had random orientation, while a large number of α’ in the equiaxed crystal region were larger, about 80 μm, and the grain orientation was more obvious. The internal structure of the different crystal regions was staggered by martensite needles, which ended at the grain boundary, forming a net basket structure.

**Figure 7:**
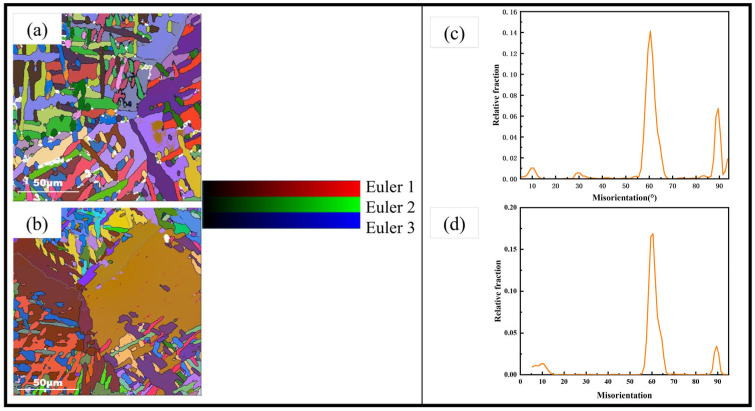
Columnar crystal region Euler diagram (**a**) and its corresponding orientation difference diagram (**c**) and equiaxed crystal region Euler diagram (**b**) and its corresponding orientation difference diagram(**d**).

The Euler diagram of the two-grain region is shown in Figure 7, it could be seen that the adjacent orientation difference in α’ grains in the two regions was distributed at 10°, 60° and 90°, and there were many distributions in the 30° columnar crystal region. This conforms to the Burgers orientation relationship of β → α transition [29].

**Figure 8:**
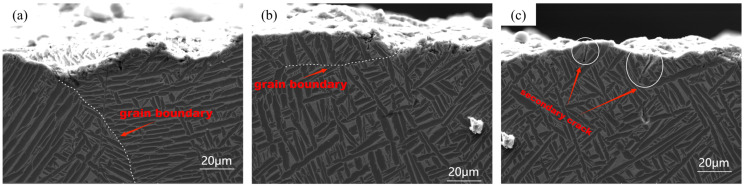
SEM diagrams of grain boundaries and secondary cracks along the crack propagation path. (a,b) Equiaxed and columnar grain boundary morphology (c) Secondary crack.

**Figure 9:**
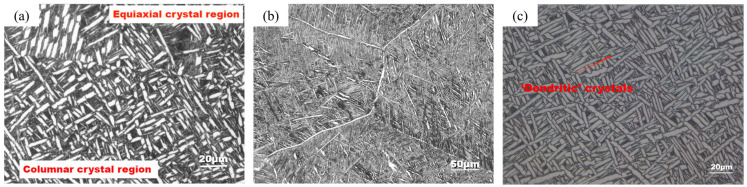
Metallographic structure diagram of the boundary area between the equiaxed crystals and columnar crystals (**a**). 200 times metallographic structure diagram (**b**). Internal metallographic structure diagram of grains (**c**).

Part of the crack propagation paths displayed under a scanning electron microscope are shown in Figure 8. On the macro scale, the T1 crack propagation path was smoother, while the T2 propagation path was more tortuous and rougher. In the propagation process, the T1 and T2 samples showed two combined propagation modes including intergranular propagation and transgranular propagation. The α’ aspect-to-aspect ratio of the crack propagation path in T2 was obviously greater than that of T1, and many secondary cracks were also produced on the propagation path. High magnification micrographs of the two-grain region are shown in Figure 9. Their deepest part was “droplet-shaped”, as shown in Figure 9(c), indicating that the surface microcracks were not formed by propagating deeper along the α grains but by the interconnection between the defects near the fracture surface and the surface damage under the influence of stress concentration. It is worth noting that both the T1 and T2 cracks initiated and propagated mainly along the columnar crystal region, especially in the T1 sample. The cracks were located at the junction of the equiaxed crystal region and columnar crystal region, but the cracks still chose to propagate mainly along the columnar crystal region. One of the reasons is that the grain distribution in the columnar crystal was messy, and the grains were short once the cracks began to propagate in the columnar crystal; this is also the reason why the crack propagation path was longer in the columnar crystal region than in the equiaxed crystal region with the same width [30]. The variable propagation path reduced the probability of the propagation edge reaching the equiaxed crystal region and extended along the columnar crystal region, which helped to improve the crack propagation resistance of the material.

### 3.3. Microscopic Mechanism of Crack Propagation

**Figure 10:**
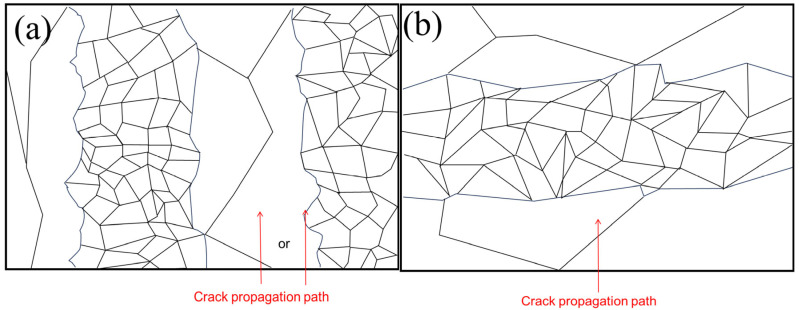
Schematic diagram of fatigue crack growth path in L-T direction (**a**) and T-L direction (**b**).

**Figure 11:**
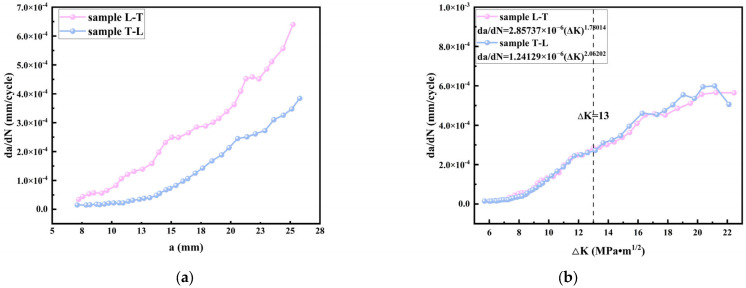
da/dN~ΔK diagram (**a**) and da/dN~a diagram (**b**) of L-T and T-L crack propagation specimens.

According to the micro-morphology of LMD TC11, the micro-morphology of the grains passing through the crack propagation in the L-T direction and the T-L direction were different. For example, the schematic diagram of the crack propagation paths in different forming directions is shown in Figure 10. The T-L sample would alternately pass through the equiaxed crystal region and columnar crystal region in turn during crack propagation, which would have a certain impact on the crack propagation rate. Paris proposed that the stress field intensity at the crack tip could be represented by the stress intensity factor K1, making it the true driving force behind crack propagation. Based on this, he formulated a crack growth equation directly related to the range of the stress intensity factor ΔK. Whether there was obvious anisotropy in the crack propagation can be studied by observing the relationship between the da/dN~ΔK (Figure 11a) and da/dN~a (Figure 11b) during crack propagation, in which there was a Paris relationship between the crack propagation rate and the variation range ΔK of the stress intensity factor [31]:

(1) $$\frac{da}{dN}=c{\left(\Delta K\right)}^{m}$$

In this study, three samples were taken in both directions, and the combined value was taken as the final fitting coefficient, as shown in Figure 11a. It can be seen from the two diagrams that LMD TC11 had certain fluctuations in the crack propagation process in all directions. In the da/dN~ΔK diagram, when ΔK < 13 Mpa m^1/2^, it showed a rapid crack propagation stage. When it exceeded this value, the propagation rate began to produce a stable propagation trend, and the distance between the two crack propagation rates was not large. From the da/dN~a relationship diagram of the different forming directions, it could be seen that the crack growth rate in the different forming directions showed certain fluctuations with the increase in the crack length, and the fluctuation in the L-T forming direction was more obvious, which was due to the increase in the crack growth rate when the crack passed through equiaxed grains with stronger grain directivity, resulting in short fluctuations. Grain boundaries act as barriers that disrupt the continuity of crack growth paths, reducing the crack propagation rate and causing deviations in the crack trajectory [32,33]. Generally speaking, there was no obvious anisotropy in the crack growth rate of LMD TC11, which indicates that the crack growth was not greatly affected by the grain orientation but mainly by the type of mesh basket structure inside the grain.

**Figure 12:**
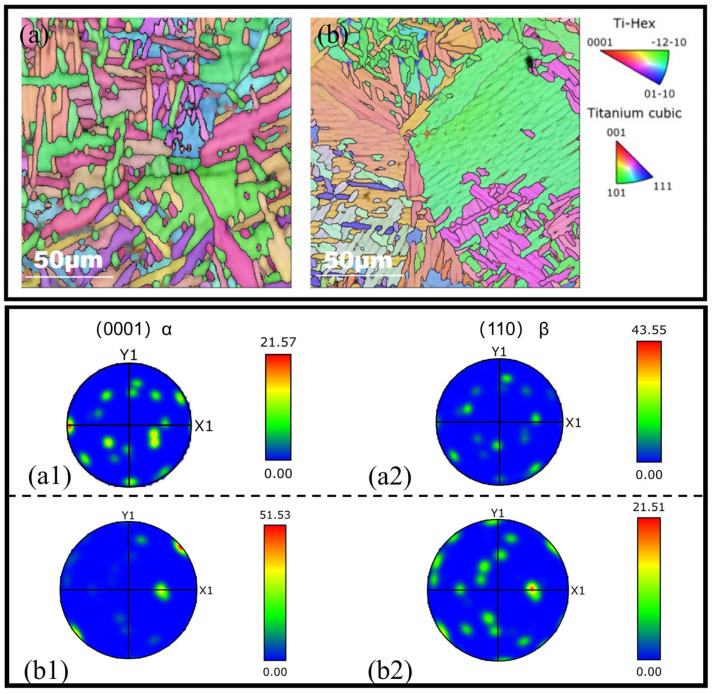
Columnar intracrystalline IPF pattern (**a**). Polar pattern α (**a1**) β (**a2**) and equiaxed intracrystalline IPF pattern (**b**). Polar pattern α (**b1**) β (**b2**).

**Figure 13:**
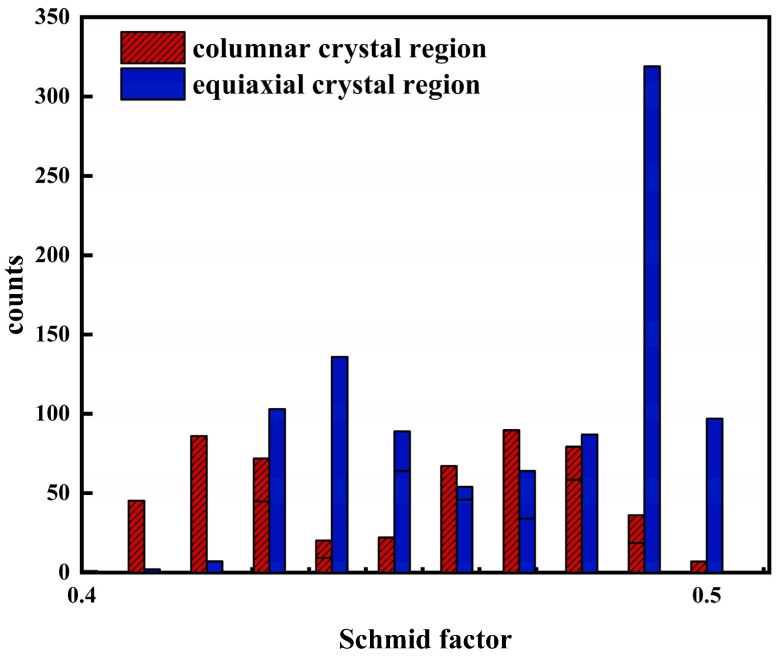
SF statistics of columnar and equiaxed crystals.

The polar diagrams of the α phase and β phase in the columnar crystal region and equiaxed crystal region are shown in Figure 12. The highest polar density of α grains in the equiaxed crystal (51.53) was much larger than that of the columnar crystal (21.57) while the highest polar density of the β crystal (21.51) was indeed much smaller than that of the columnar crystal (43.55). During the LMD forming process, the initial β columnar grains grew through several consecutive layers, showing a strong {001}β preferred direction along the deposition direction. The α phase inherited the orientation relationship of the previous β grains [34], so as shown in Figure 12, these inherited grain orientations still satisfied {0001}α//{110}β<111>β//[11$\bar{2}$0] α, which followed the Burgers orientation relationship. The β-crystal texture in the columnar crystals was more obvious, while the α-crystal texture in the equiaxed crystals was more obvious. The crystal orientation would cause cyclic plastic deformation, such as the slip of grains near the crack source under the action of load, thus affecting the fatigue life. The heterogeneous distribution of the crystal orientations produced a variety of crystallographic textures, further leading to different difficulty levels of slip [35]. One of the important elements to determine whether the slip could be activated was the Schmid factor required for the slip. The SF statistical diagram of the two crystal regions is shown in Figure 13. There were five slip systems that could be activated in HCP, while the slip direction in α-Ti was mainly {11$\bar{2}$1}<11$\bar{2}$0>. The slip direction in β-Ti was {111}<110>. The statistical results of the SF show that there was no difference in the average value of the slip system between the two crystal regions, but when the Schmid factor level was 0.4–0.5, the frequency of the basal slip system in the equiaxed crystal region was higher than that in the columnar crystal region. This is also the reason why the crack propagation path propagated rapidly through the equiaxed crystal region, which made the propagation rate fluctuate.

**Figure 14:**
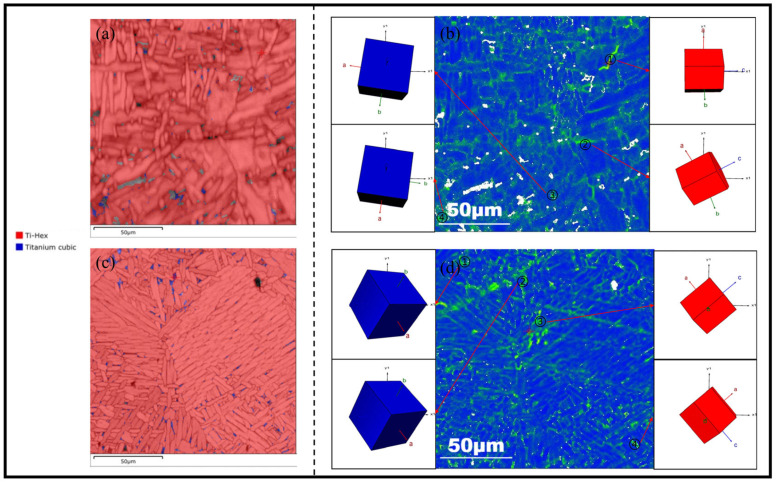
Columnar crystal phase distribution diagram (**a**). KAM part hcp crystal bcc crystal orientation diagram (**b**) and equiaxed crystal phase distribution diagram (**c**). KAM part hcp crystal bcc crystal orientation diagram (**d**).

The distribution and local orientation difference map (KAM) of the phase structure in the columnar crystals and the equiaxed crystals were as follows (Figure 14). The KAM map reflected the degree of plastic deformation to a certain extent. Dislocation accumulation generally appeared at the α/β boundary, and the degree of plastic deformation at the junction of the trigeminal grain boundary was larger and more concentrated than that in the crystal. The strength and plasticity of the TC11 LMD mainly stemmed from the fact that the grain boundary density was affected by the grain morphology [36,37]. A similar observation was made in recent studies on metastable austenitic steels, where the dislocation density and accumulation were found to increase at the grain boundaries, particularly under asymmetric cyclic loading conditions. This accumulation of dislocations was closely tied to the fatigue behavior, as higher dislocation density near the grain boundaries could enhance the work hardening effect, potentially increasing the material’s resistance to crack propagation in certain loading regimes [38]. Under the influence of α’ orientation and β phase content and distribution, the two different crystal regions showed different concentrations of plastic deformation. In the columnar crystal region with random α grain distribution, the plastic deformation was also scattered and uniform. In the equiaxed crystal region with the obvious directional distribution of α grains, the degree of plastic deformation between the large number of parallel grains was less than that at the α/β grain boundary and the two larger orientation α grain boundary, which indicated that the dislocation accumulation in this region was less concentrated and dispersed, and the degree of plastic deformation was very uneven. That is to say, the crack growth was accelerated when the slip zone was parallel to the α/β grain boundary, while the plastic deformation in the two phases was slightly different. However, the plastic deformation in the two phases was slightly different. The plastic deformation distribution caused by these microscopic dislocation accumulations well explains why crack propagation was easy to propagate in the columnar crystal region.

## 4. Conclusions

In this paper, the fatigue fracture properties, plastic deformation, and crack propagation behavior of the TC11 formed by LMD after double annealing treatment were studied under a special bimodal microstructure. According to the current research results, the following conclusions were drawn:

1. In the fatigue tensile process of LMD TC11, the surface and subsurface pores were mostly used as crack sources. As the crack source, the position of the pores could affect the cracking more than the size of the pores. In addition, the toughness of the material became one of the factors that affected fatigue life, which could affect the stable expansion of the test process and the mode of fracture stage. By addressing the pore formation during the LMD process, such as through better control of the laser parameters or optimizing powder feed, it may have been possible to reduce the fatigue-related failures in the critical components like aerospace and automotive parts. Furthermore, the toughness of the material, influenced by pore distribution and microstructure, directly affected the stable crack propagation during testing, suggesting that the material toughness must be considered in tandem with pore control for improved fatigue performance;
2. After heat treatment, the specimen showed a special bimodal microstructure, that is, the columnar crystals and equiaxed crystals were alternately arranged, and different internal microstructures led to a certain fluctuation in macroscopic crack propagation. SF statistics showed that the frequency of the substrate slip system in the equiaxed crystals was higher than that in columnar crystals when the slip direction β-Ti{111}<110>SF level was 0.4–0.5, which led to an accelerated crack propagation rate in the equiaxed crystals, but this did not make the specimens with different forming directions. This insight is crucial for applications where material durability is essential, such as in aerospace and biomedical implants;
3. Both the α’ and β crystals in the equiaxed and columnar regions satisfied the Burgers orientation relation ({0001}α//{110}β). It was found that cracks tend to propagate along columnar regions when nucleating near equiaxed crystals. The reason was that the dislocations were more dispersed in the columnar regions and more concentrated in the equiaxed regions in the KAM diagram, so there were more path choices in the columnar crystals at the initial stage of crack propagation and the probability of propagation along the columnar crystals was greater. Understanding the microstructural behavior at the grain boundaries and optimizing the grain size distribution could provide effective strategies to improve the fatigue life of LMD titanium alloys in high-performance applications, such as aerospace components and biomedical devices.

To improve fatigue properties, reducing pore size and optimizing the distribution of pores during the LMD process would be key. Post-processing methods like hot isostatic pressing (HIP) could also be explored to mitigate porosity-related fatigue degradation. Future studies could focus on optimizing annealing parameters to better control the formation of equiaxed and columnar grains, with the aim of achieving a more uniform microstructure to improve overall fatigue resistance. This insight suggests that controlling the grain boundary structure and dislocation distribution in columnar regions could be an effective strategy to improve crack resistance. Future research could investigate the effects of grain boundary engineering and other advanced heat treatment techniques to further enhance the fatigue life of LMD titanium alloys.

## Figures and Tables

**Table 1:** Prefabricated titanium alloy powder composition.

Powder Ingredients									
**C**	**O**	**N**	**H**	**Al**	**Mo**	**Si**	**Fe**	**Zr**	**Ti**
0.012	0.11	0.013	0.002	6.46	3.46	0.26	0.12	1.73	matrix

**Table 2:** Quantitative statistic of secondary cracks.

Nf/×10^3^ d/μm	28.1	82.4	106.9	297
346	0	0	1	1
1038	0	2	16	3
1730	2	10	42	36
2422	3	41	57	52
3114	13	56	65	66
3806	35	43	33	57
4498	11	36	20	39
5190	10	15	12	16
5882	5	4	4	13
6574	0	2	3	2
7266	0	0	0	0
Total	79	209	253	285

## Data Availability

The original contributions presented in this study are included in the article. Further inquiries can be directed to the corresponding author.
